# Evolutionarily significant units of the critically endangered leaf frog *Pithecopus ayeaye* (Anura, Phyllomedusidae) are not effectively preserved by the Brazilian protected areas network

**DOI:** 10.1002/ece3.3261

**Published:** 2017-09-20

**Authors:** Rafael Félix de Magalhães, Priscila Lemes, Arley Camargo, Ubirajara Oliveira, Reuber Albuquerque Brandão, Hans Thomassen, Paulo Christiano de Anchietta Garcia, Felipe Sá Fortes Leite, Fabrício Rodrigues Santos

**Affiliations:** ^1^ Programa de Pós‐Graduação em Zoologia Instituto de Ciências Biológicas Universidade Federal de Minas Gerais – UFMG Belo Horizonte Minas Gerais Brasil; ^2^ Programa de Desarrollo Universitario Centro Universitario de Rivera Universidad de la República – UdelaR Rivera Uruguay; ^3^ Laboratório de Herpetologia Departamento de Zoologia Instituto de Biociências de Rio Claro Universidade Estadual “Júlio Mesquita Filho” – UNESP Rio Claro São Paulo Brasil; ^4^ Centro de Sensoriamento Remoto Instituto de Geociências Universidade Federal de Minas Gerais – UFMG Belo Horizonte Minas Gerais Brasil; ^5^ Laboratório de Fauna e Unidades de Conservação Departamento de Engenharia Florestal Faculdade de Tecnologia Universidade de Brasília – UnB Brasília Distrito Federal Brasil; ^6^ Graduação em Ciências Biológicas Instituto de Ciências Biológicas Universidade Federal de Minas Gerais – UFMG Belo Horizonte Minas Gerais Brasil; ^7^ Laboratório Sagarana Instituto de Ciências Biológicas e da Saúde Universidade Federal de Viçosa – UFV Florestal Minas Gerais Brasil

**Keywords:** approximate Bayesian computation, *campos rupestres*, conservation genetics, ecological niche modeling, niche overlap, statistical phylogeography

## Abstract

Protected areas (PAs) are essential for biodiversity conservation, but their coverage is considered inefficient for the preservation of all species. Many species are subdivided into evolutionarily significant units (ESUs) and the effectiveness of PAs in protecting them needs to be investigated. We evaluated the usefulness of the Brazilian PAs network in protecting ESUs of the critically endangered *Pithecopus ayeaye* through ongoing climate change. This species occurs in a threatened mountaintop ecosystem known as *campos rupestres*. We used multilocus DNA sequences to delimit geographic clusters, which were further validated as ESUs with a coalescent approach. Ecological niche modeling was used to estimate spatial changes in ESUs’ potential distributions, and a gap analysis was carried out to evaluate the effectiveness of the Brazilian PAs network to protect *P. ayeaye* in the face of climate changes. We tested the niche overlap between ESUs to gain insights for potential management alternatives for the species. *Pithecopus ayeaye* contains at least three ESUs isolated in distinct mountain regions, and one of them is not protected by any PA. There are no climatic niche differences between the units, and only 4% of the suitable potential area of the species is protected in present and future projections. The current PAs are not effective in preserving the intraspecific diversity of *P. ayeaye* in its present and future range distributions. The genetic structure of *P. ayeaye* could represent a typical pattern in *campos rupestres* endemics, which should be considered for evaluating its conservation status.

## 1 INTRODUCTION

Protected areas (PAs) are the cornerstone conservation strategy to maintain viable populations (Watson, Dudley, Segan, & Hockings, 2014). The creation and maintenance of PAs should consider not only their actual coverage, but also their effectiveness in future species survival. In practice, the global network of PAs is inefficient in representing biodiversity, including threatened species (Nori et al., 2015; Rodrigues et al., 2004). Most PAs’ policies consider biological diversity at the species level or above (Gaston, Jackson, Cantú‐Salazar, & Cruz‐Piñón, 2008; Geldmann et al., 2013), which are related to the phylogenetic and ecological dimensions of biodiversity. The intraspecific dimension, which is related to the evolutionary potential of organisms, is an essential aspect in the planning of conservation policies, especially for endangered species (Bowen & Roman, 2005). Nevertheless, intraspecific diversity is frequently neglected in conservation planning, even though adaptive potential and stress resistance are positively correlated with genetic diversity (Bálint et al., 2011; Pauls, Nowak, Bálint, & Pfenninger, 2013).

An efficient PAs network design should be planned considering species viability toward the future, taking climate disruptions into account. From a long‐term perspective, climate changes may worsen the effectiveness of existing PAs in the future, because PAs are static while species’ ranges are expected to shift spatially (Lemes, Melo, & Loyola, 2014; Monzón, Moyer‐Horner, & Palamar, 2011). This hypothesis is reinforced by evidence that many species’ distributions have already been modified by climate change in plants, arthropods and vertebrates (Chen, Hill, Ohlemüller, Roy, & Thomas, 2011; Parmesan & Yohe, 2003), including altitudinal shifts recorded recently for anurans from South Africa and Indonesia, for example (Boots, Erasmus, & Alexander, 2015; Kusrini et al., 2017). From a population viewpoint, a species is not always a single unit for conservation because intraspecific subunits may evolve independently in response to climate disruptions (Bálint et al., 2011; Forester, DeChaine, & Bunn, 2013). Due to these reasons, current assessments about the future effectiveness of PAs could be over‐optimistic (Pauls et al., 2013). Therefore, an effective plan for future species viability should incorporate the intraspecific levels of biodiversity. In this context, evolutionarily significant units (ESUs) represent ideal targets for conservation because they contain the raw material for future evolutionary adaptations (Bowen & Roman, 2005; Fraser & Bernatchez, 2001; Pauls et al., 2013). Thus, population viability evaluations should begin with the challenging task of delineating these intraspecific units (Fraser & Bernatchez, 2001).

The conservation status of amphibians is alarming in this context because they are among the most threatened vertebrates (Pimm et al., 2014) and many species are suffering population declines associated with a variety of threats, including climate change (Blaustein et al., 2011; Stuart et al., 2004). Despite these serious conservation concerns, 42% of the amphibian richness is misrepresented or completely outside PAs (Nori et al., 2015). Amphibians generally exhibit high levels of genetic structuring usually associated with the geographic isolation of demes (Allentoft & O'Brien, 2010; Rodríguez et al., 2015), which makes it difficult to assess whether the differentiated lineages are either distinct species or populations (e.g., Carnaval & Bates, 2007; Gehara, Canedo, Haddad, & Vences, 2013). In these cases, if conservation policies do not consider lineage differentiation, a significant loss of cryptic diversity is expected under climate change (Bálint et al., 2011; Pauls et al., 2013). For this reason, understanding intraspecific ESUs, their evolutionary history, spatial distribution, and connectivity are important steps toward minimizing genetic diversity loss among isolated amphibian populations (Bálint et al., 2011; Beebee & Griffiths, 2005; Pauls et al., 2013; Weeks, Stoklosa, & Hoffmann, 2016).

**Figure 1:**
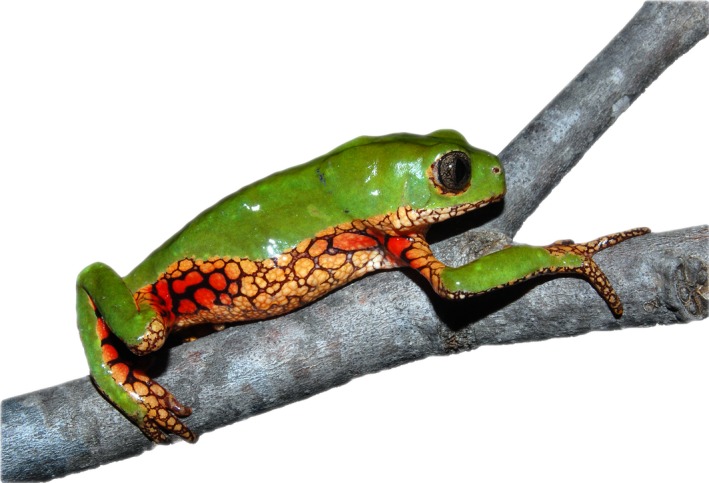
An individual of *Pithecopus ayeaye* B. Lutz, 1966 from type locality, Morro do Ferro, Poços de Caldas—MG. Photograph by RAB

The reticulated leaf frog *Pithecopus ayeaye* B. Lutz, 1966 (Anura, Phyllomedusidae) (Figure 1) is a high‐altitude endemics leaf frog of southeastern Brazil, which reproduces throughout the rainy season (from October to January) (Oliveira, 2017). The species is rare and shows low individual density in most of its range (Araujo, Condez, & Haddad, 2007; Baêta, Caramaschi, Cruz, & Pombal, 2009). It produces clutches with few large eggs and the larvae generally inhabit rocky stream pools, with crystal clear and slow‐flowing water (Oliveira, 2017; Pezzuti, Leite, & Nomura, 2009). The males are territorials, defending pulleys along streams with riparian vegetation for oviposition where funnel nests are constructed above the water using the leaves (Nali, Borges, & Prado, 2015; Oliveira, 2017). These leaves are not randomly chosen, but those from Melastomataceae bushes on the margins of the rivulets are preferred (Oliveira, 2017), probably due to foliar area that minimizes the risk of clutch desiccation and the presence of spiny structures in leaves for anchoring the eggs (Dias, Maragno, Prado, & Cechin, 2014; Oliveira, 2017). When tadpoles hatch from eggs, they fall into the water and complete their development (Dias et al., 2014). The species is susceptible to stochastic climatic events, like dry periods in the rainy season that can cause severe stream drought and dehydration of egg clutches.

*Pithecopus ayeaye* is classified as critically endangered (CR) by the International Union for Conservation of Nature (IUCN; Caramaschi, Cruz, Lima, & Brandão, 2010) based on B1ab(iii)+2ab(iii) criteria, as it was only known from two disjunct localities threatened by severe habitat loss due to mining and human‐induced fires (Caramaschi et al., 2010). The threat sources are more numerous because the mountain grasslands where *P. ayeaye* occurs are also menaced by forestry, cattle farming, nonsustainable “ecotourism,” and poorly planned urbanization (Silveira et al., 2016). These threats impact directly or indirectly on the highland streams where *P. ayeaye* breeds. Hence, *P. ayeaye* was included as a priority in a Brazilian national action plan for Espinhaço Range herpetofauna (ICMBio, 2011). After the discovery of new localities (Araujo et al., 2007; Baêta et al., 2009), the species no longer meets the geographic requirements to be categorized as CR. For this reason, *P. ayeaye* was recently removed from all threat categories in the Brazilian List of Endangered Species (ICMBio, 2014), and excluded from the national agenda of conservation priorities. However, the impacts of the fragmented distribution of the species on its genetic diversity and adaptive potential were not considered in the current categorization.

*Pithecopus ayeaye* occurs in a threatened ecosystem (Fernandes, Barbosa, Negreiros, & Paglia, 2014) and exhibits a naturally fragmented distribution in mountaintops of southeastern Brazil. Although IUCN informs that the species occurs at elevations over 647 m a.s.l. (Caramaschi et al., 2010), probably due to a misunderstanding of Araujo et al. (2007)'s description of Furnas do Bom Jesus State Park limits, literature and collection data suggest that the distribution is mainly restricted at elevations higher than 900 m a.s.l. (Table S1). It occurs mainly in the southern limits of the *campos rupestres* grasslands (see Silveira et al., 2016), but it is also found in grassland patches of the Poços de Caldas Plateau (Araujo et al., 2007; Baêta et al., 2009). The *campos rupestres* are ecosystem patches characterized by extensive quartzitic, arenitic, or ironstone outcrops (Silveira et al., 2016) with typical streams that *P. ayeaye* uses for reproduction. These areas are part of the Brazilian Shield sky islands complex (*sensu* Warshall, 1995) and are on the border between the Cerrado and Atlantic Forest domains, two biodiversity hotspots (Myers, Mittemeier, Mittemeier, Fonseca, & Kent, 2000). The *campos rupestres* is a megadiverse ecosystem with high endemism rates in a variety of organisms (e.g., Chaves, Freitas, Vasconcelos, & Santos, 2015; Jacobi, Carmo, Vincent, & Stehmann, 2007; Silveira et al., 2016), including anurans (Leite, Juncá, & Eterovick, 2008). Although it is distributed in <1% of the Brazilian land surface, the *campos rupestres* contains about 15% of the vascular plant richness catalogued for the country (Silveira et al., 2016). Nevertheless, it has been largely neglected in Brazilian research and conservation policies (Jacobi et al., 2007; Silveira et al., 2016), even though there is clear evidence of an ominous future for this unique mountaintop ecosystem. For example, a climatic model has predicted a loss of 95% of suitable areas, in an optimistic scenario, by the year 2080 (Fernandes et al., 2014).

There have been few phylogeographical studies of the *campos rupestres* biota, but all show strong genetic structuring between sky island populations (e.g., Bonatelli et al., 2014; Collevatti, Rabelo, & Vieira, 2009; Freitas, Chaves, Costa, Santos, & Rodrigues, 2012), suggesting that cryptic spatial diversification is common for this endemic biota. This intraspecific diversification has been frequently used to identify conservation units within species, which have been applied in the definition of Evolutionarily Significant Units (ESUs). Moritz (1994) used phylogeographical analysis based on sequence markers, defining defined ESUs as reciprocally monophyletic, mitochondrial DNA (mtDNA) groups with significant divergence in nuclear alleles frequencies among them. The advantages of this criterion are its objectivity and generality, applicable to phylogeographical data (Fraser & Bernatchez, 2001), but it might not be able to distinguish ESUs with incomplete lineage sorting (ILS) or mtDNA gene flow. To accommodate these genealogical processes, Fraser and Bernatchez (2001) proposed that ESUs are intraspecific lineages with highly restricted gene flow among them, allowing ESUs delimitation without reciprocal monophyly.

In this study, we aimed to delimit ESUs within *P. ayeaye* using a broad geographic sampling and multilocus sequence data in a statistical phylogeography framework coupled with GIS information (e.g., Forester et al., 2013). We analyzed multiple island models to validate ESUs, taking ILS and gene flow into account, and used niche divergence tests to refine predictions about the effectiveness of PAs in preserving diversity at the present and in the future (Beerli & Palczewski, 2010; Carstens, Brennan, et al., 2013; Warren, Glor, & Turelli, 2008). In order to evaluate the future survival of distinct ESUs in the current PAs, we used ecological niche modeling (ENM) to project ESUs’ ranges based on the ongoing and future scenarios of climate change (Bálint et al., 2011), applying rigorous model evaluation to deal with the scarcity of occurrence data due to the rarity of the species (Shcheglovitova & Anderson, 2013). From the available evidence, we expect a spatially associated intraspecific diversification for *P. ayeaye* and the loss of suitable areas for the species is expected in a future scenario of climate change. Here we assume that the geographic distribution of the species is partially explained by climate, as the areas where *P. ayeaye* occurs are islands of subtropical climate in a tropical matrix (Alvares et al., 2013), which could favor a set of physiological and/or behavioral adaptations.

## 2 MATERIAL AND METHODS

### 2.1 Data collection

**Figure 2:**
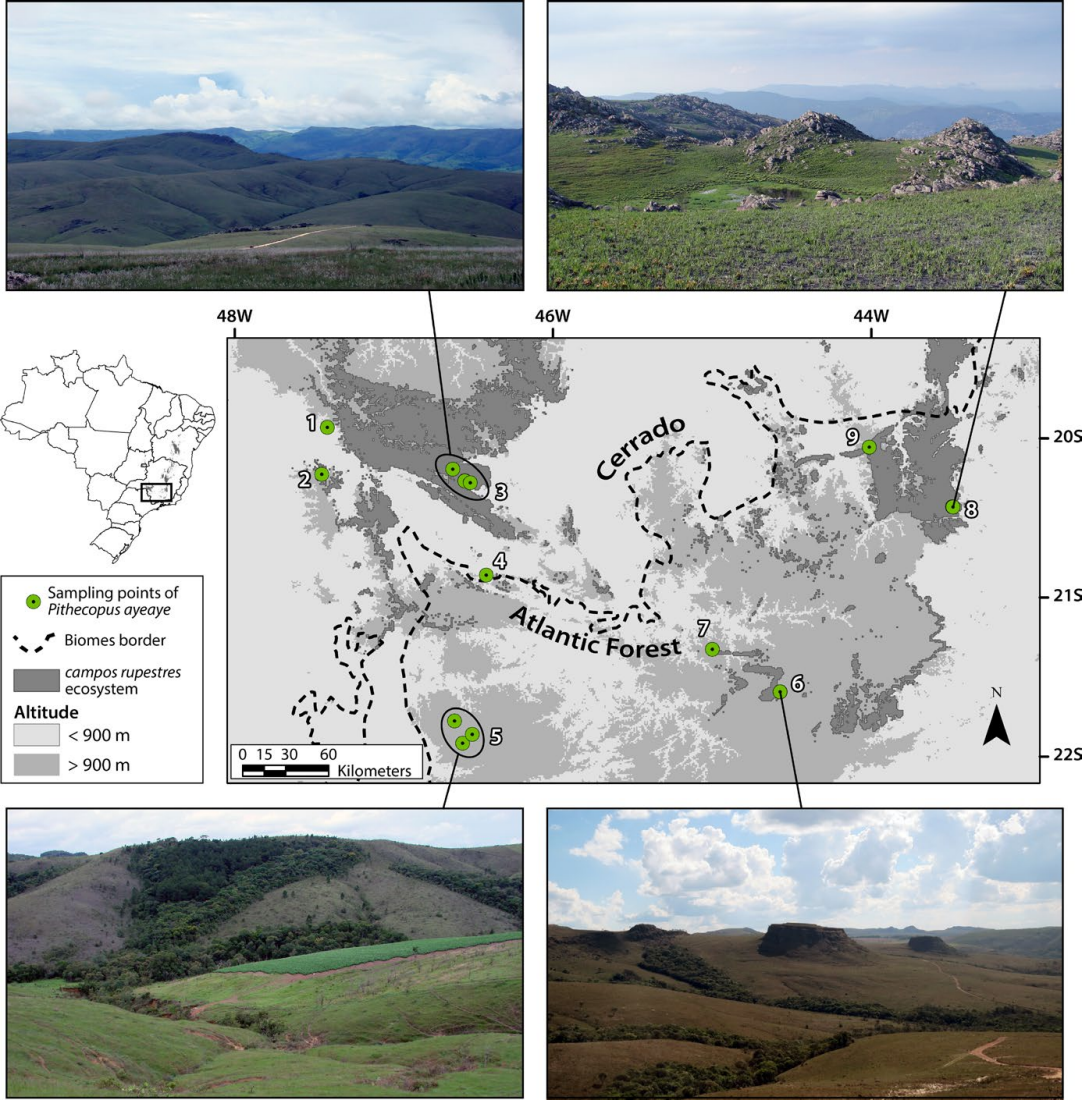
Map of sampled locations of *Pithecopus ayeaye* B. Lutz, 1966 with some examples of landscapes where the species lives. 1. Serra da Canastra—Sacramento, MG; 2. Furnas do Bom Jesus State Park—Pedregulho, SP; 3. Region of the National Park of Serra da Canastra—São Roque de Minas, MG (photograph by Tiago L. Pezzuti); 4. Serra da Ventania—Alpinópolis, MG; 5. Poços de Caldas Plateau—Poços de Caldas, MG (photograph by RAB; note the erosion on the creek slope); 6. Chapada das Perdizes—Minduri, MG (photograph by RFM); 7. Serra do Campestre—Lavras, MG; 8. Serra de Ouro Preto—Ouro Preto, MG (photograph by Tiago L. Pezzuti); 9. Serra da Moeda—Nova Lima, MG. The limits of *campos rupestres* ecosystem were modified from Fernandes et al. (2014)

We obtained tissue samples mainly from museums and collections, many of them with voucher specimens (see Appendix S1). These were complemented with samples collected by us from a few other locations to bring our total to 88 individuals from 13 sample points in nine counties (Figure 2, Table S1), which is a significant sampling in relation to known species geographic distribution (see Baêta et al., 2009). In the case of tadpole sampling, we excluded individuals in similar developmental stages collected together in the same place or stored in the same museum lot, in order to avoid first‐degree relatives and, consequently, to maximize sampling randomness. We extracted genomic DNA from liver or muscle samples using the phenol‐chloroform protocol (Sambrook & Russel, 2001). For all specimens, we amplified and sequenced an 896‐bp fragment of the mitochondrial Cytochrome b (Cyt‐b) (primers: MVZ15, Moritz, Schneider, & Wake, 1992; Cyt‐b‐ARH, Goebel, Donnelly, & Atz, 1999), a nuclear 601‐bp exon fragment of proopiomelanocortin (POMC) (primers POMC‐1 and POMC‐2, Wiens, Fetzner, Parkinson, & Reeder, 2005), and a nuclear 518‐bp intron 5 fragment of ribosomal protein L3 (RPL3) (primers RPL3intF and RPL3intR, Pinho et al., 2010). Polymerase chain reactions (PCRs) were performed in a 15 μl reaction volume containing: 20 ng of genomic DNA, 1× buffer, 2.5 mmol/l MgCl_2_, 1.25 μmol/l each primer, 3 mmol/l dNTPs, 0.72 μg bovine serum albumin, and 0.625 U Platinum^™^ *Taq* DNA polymerase (Thermo Fisher Scientific). Amplifications were performed as one initial denaturation for 95°C during 5 min followed by 35 cycles [denaturation at 95°C for 30 s, variable melting temperatures and times between fragments (54°C by 40 s to Cyt‐*b*; 60°C by 50 s to POMC; and 62°C by 40 s to RPL3), extension at 72°C for 1 min/1,000 bp] and a final extension at 72°C for 7 min. We reduced temperature melting, increased time melting, and/or increased up to 3.5 mmol/l MgCl_2_ for samples difficult to amplify. All single PCR products were purified using a Sambrook and Russel's (2001) polyethylene glycol 20% protocol, with some modifications (Santos Júnior, Santos, & Silveira, 2015).

Purified amplicons were fluorescence‐marked through bigdye^™^ terminator v3.1 cycle sequencing kit (Thermo Fisher Scientific) following the manufacturer's instructions. The primers used in this reaction were the same to those used in PCRs, except for RPL3‐intF (Pinho et al., 2010), which was replaced with RPL3‐P3 (5′ WCTGGCCTGCTCTGGTTAT 3′) designed by us in Primer3Plus (Untergasser et al., 2007). The marked amplicons were analyzed in an automatized ABI 3130xl DNA sequencer (Thermo Fisher Scientific) in both directions.

### 2.2 Sequence edition and data characterization

Sequence fluorograms were interpreted, assembled, pre‐aligned, and edited in seqscape ^™^ 2.6 (Thermo Fisher Scientific). Edited fragments were aligned with the clustalW module of the mega7 software (Kumar, Stecher, & Tamura, 2016; Larkin et al., 2007), with gap opening penalized 10 times more than extension for intron alignment. The gametic phases of nuclear markers were reconstructed through the algorithm implemented in phase 2.1.1 software (Stephens, Smith, & Donelly, 2001), which were interconverted to fasta format using seqPHASE web tool (Flot 2010). In cases where more than one haplotype pair was reconstructed, we selected the more probable pair for subsequent analyses, except those based in genealogies reconstruction. In these cases, haplotype phases not fully resolved (PP < .9) were checked by eye and the uncertain nucleotide positions were kept. Because there were some heterozygous individuals for insertions and deletions in RPL3, we estimated their haplotypic phases via the codification of superimposed traces in indelligent 1.2 web‐based software (Dmitriev & Rakitov, 2008). After these steps, we selected the best fit model of molecular evolution among 88 substitution schemes using the BIC criterion in jmodeltest 2.1.10 software (Darriba, Taboada, Doallo, & Posada, 2012) for each DNA fragment.

We did a maximum likelihood (ML) test of the strict molecular clock for each DNA fragment under the same substitution model selected in the previous step as implemented in mega7 (Kumar et al., 2016). Because an input tree is required for this test, we generated gene trees of unique haplotypes in RaxML 8.2.4 software (Stamatakis, 2014), choosing the “best scoring ML‐tree with rapid bootstrap” option under the GTRCAT model. The null hypothesis of equal evolutionary rate along the tree was not rejected for both Cyt‐b (*p* = .85) and POMC (*p* = .47), but it was rejected for RPL3 (*p* < .001). We applied this test to build simpler and less parametric models in subsequent analyses, aiming to achieve a balance between bias and variance in results (Kelchner & Thomas, 2006).

### 2.3 ESUs discovery, diversity, and relationships

We applied a two‐step ESU delimitation consisting of sequential stages of discovery and validation, which is analogous to the rationale proposed by Carstens, Pelletier, Reid, and Satler (2013) for species delimitation, but accommodating gene flow explicitly. For the ESUs discovery, we first tested spatial population structure using the geneland package in r (Guillot, Estoup, Mortier, & Cosson, 2005a; Guillot, Mortier, & Estoup, 2005b; R Core Team 2016). We chose a spatially explicit model because of our expectation of structure is related to isolation in sky islands. The mtDNA data were entered as haplotypes, while the nDNA haplotypes were encoded as alleles for the analysis. Because our goal was to identify those lineages that minimized global gene flow (Fraser & Bernatchez, 2001), we opted for the uncorrelated allele frequencies model, which is generally unable to detect subtle structuring (Guillot, 2008). We made ten parallel runs with 1 × 10^6^ iterations each. The analysis iterations each, with a thinning of 1 × 10^3^, and *K* varying between 1 and 10 biogeographical units (BUs), which we consider as being putative ESUs. Additionally, we assessed the isolation by distance (IBD) hypothesis to verify whether genetic diversity can be explained by geographic distance between localities. For this, we performed a Mantel test between log_10_‐transformed geographic and genetic distances matrices of our 13 sample points in ibdws (Jensen, Bohonak, & Kelley, 2005), with 10,000 randomizations. The multigenic distance matrix was calculated in pofad (Joly & Bruneau, 2006) using the genpofad algorithm (Joly, Bryant, & Lockhart, 2015).

To estimate the relationship among discovered BUs, we constructed a lineage tree (=species tree) in the starbeast2 (Bouckaert et al., 2014; Ogilvie, Bouckaert & Drummond, in press) using a Yule prior with a constant population model. To estimate divergence times, we used an uncorrelated log‐linear clock model for RPL3, and strict clock models for POMC and Cyt‐*b*. In the latter case, we used a standard mtDNA substitution rate (mean of 0.01 substitutions per lineage per million years; Johns & Avise, 1998), due to the lack of fossils for calibration. This is similar to the ND2 rate (Crawford 2003), which is widely used in amphibian dating (e.g., Carnaval & Bates, 2007). This analysis was made with two replicates, with a pre‐burn‐in of 2.5 × 10^7^ followed by 7.5 × 10^7^ iterations each. The analysis was repeated twice, and we checked for convergence, stationarity, and minimum adequate effective sampling size (ESS > 200) of analyses in tracer v1.6 (Rambaut, Suchard, Xie, & Drummond, 2014). Furthermore, we built statistical parsimony networks in popART 1.7 software (Leigh & Bryant, 2015; Templeton & Sing, 1993) to visualize the relationships between the haplotypes of each gene fragment. Finally, we estimated global and BU‐specific summary statistics for each locus in dnaSP (Librado & Rozas, 2009), including unique haplotype numbers (h), haplotype (Hd) and nucleotide (π) diversities, and Tajima's D (Tajima, 1989).

### 2.4 ESUs validation

We estimated the historical gene flow (effective number of migrants, *M *=* 4N*_*e*_ *m*) and the genetic diversity (*ϴ *= *4N*_*e*_μ) of the populations under a coalescent framework using migrate‐n (Beerli, 2006). We first implemented a Bayesian full model accounting for the BUs (Fig. S1, model 1) using an empirical transition‐transversion ratio (R_Cyt‐*b*_ = 4.661; R_RPL3_ = 1.154; R_POMC_ = 1.449) estimated under the K80 model in mega7 (Kimura, 1980; Kumar et al., 2016), and a scheme of relative mutation rates among loci. Individuals with missing data in haplotypes were excluded, as this may result in spurious results in migrate‐n (Carstens, Brennan et al., 2013). Initial values for parameters were derived from F_ST_ estimates. We conducted the analysis with two parallel runs, using a static heat strategy, setting one long and 12 short chains, with the cold chain equal to one, the hottest chain equal to 5 × 10^5^ and values of the remaining chains growing at a cumulative exponential scale of x^1.4^, starting from 1.5. We made a previous burn‐in of 1.25 x 10^6^ generations, and 2.5 × 10^6^ states were visited in each run, with a thinning of 100. Exploratory analyses with the full model allowed us to determine the sampling window for *ϴ* and *M*, and we assumed a normal distribution for these parameters.

Based on the geneland and starbeast2 results, we generated a set of seven reduced models to validate the putative ESUs assignment (Fig. S1, models 2–8). Fraser and Bernatchez (2001) advocated the use of criteria that “provide evidence of lineage sorting through highly reduced gene flow,” but they did not propose a cutoff level of gene flow for this criterion. Therefore, we tested scenarios of BU independence against scenarios of split between sister BUs, and split of all BUs (Fig. S1). In summary, we tested models ranging between all BUs forming a single ESU, and each BU as an independent ESU, with distinct routes of gene flow among them. If the gene flow is so high that two or more BUs form a single genealogically cohesive cluster, the BUs are collapsed into a single ESUs. In order to reduce the potential set of hypotheses, they were constructed under the following assumptions: (1) when present, gene flow is always bidirectional, and (2) sister BUs have gene flow between them, except in isolation models (see Fig. S1 for a graphical representation). The second assumption was based on the expectation that gene flow tends to decrease with longer splitting times between lineages (Pinho & Hey, 2010). Besides that, we take into account the nearest shared node from the lineage tree to collapse the BUs. We calculated the marginal likelihood of each model and compared them under a Bayes factor test using Bezier's approximation score (Beerli & Palczewski, 2010). Under this approach, we can select the model with the highest probability of fitting our data. We used model averaging for the final estimates of *ϴ* and *M*, and evaluated the performance of MCMC sampling through ESS and acceptance ratio values.

### 2.5 Parameters estimation through Approximate Bayesian Computation (ABC)

Because starbeast2 analyses do not take into account potential gene flow, and consequently, it can underestimate divergence time (τ) among ESU (Pinho & Hey, 2010), we also implemented an Approximate Bayesian Computation (ABC) approach to co‐estimate demographic parameters based on the lineage tree topology and the gene flow routes obtained from the best island model of ESUs. The older the lineage divergence, greater lineage sorting is expected (Maddison, 1997). For this reason, the aim of the tree dating analysis was to verify the congruence between the selected models and the time of ESU's diversification. We simulated 1 × 10^6^ coalescent genealogies for three loci with ms (Hudson, 2002), and processed them to obtain the following summary statistics in the msSS.pl script (Takebayashi, 2011): average nucleotide pairwise distances per locus (π), number of segregating sites (S), Tajima's *D* (Tajima, 1989), π within populations, and π between populations. We used uniform prior distributions for all parameters including *ϴ* per locus (lower bound: 0.01, upper bound: 10), divergence times in 4*N*_*e*_ units (0.0001, 0.5), and migration rates in 4*N*_*e*_ *m* units (0, 50). The same summary statistics were calculated for the empirical data globally, and for each ESU and locus in dnaSP (Table 1, Table S2). DNA divergence between ESUs (π between populations) was calculated as the average number of nucleotide substitutions per site between populations (Nei, 1987) for each locus in dnaSP (Librado & Rozas, 2009) (Table S2). To approximate posterior distributions of parameters, we analyzed simulated and observed summary statistics with the r package abc (Csilléry, François, & Blum, 2012) using nonlinear local regression (neural‐network algorithm) and a tolerance of 0.0002 (to retain 200 simulations). In order to evaluate the model fit and adequacy, we performed a principal component analysis (PCA) of all summary statistics from the prior, the posterior, and the empirical data with the stats package of R. Finally, we used the estimated mean substitution rates of each fragment obtained from starbeast2 to convert the ESUs split times from coalescent units to number of generations.

**Table 1:** Global and population summary statistics for sampled loci. Parameters shown are the total number of haplotypes (N), number of unique haplotypes (h), haplotype diversity (Hd), and nucleotide diversity per site (π). Tajima's *D* values were explained by chance

Locus	Model	Unit	*N*	Segregating sites	Tajima's *D*	h	Hd (*SD*)	π (*SD*)
Cyt‐*b*	HKY	Total	86	26		22	0.926 (0.012)	0.00393 (0.00021)
		Canastra	35	19	−1.037	12	0.862 (0.035)	0.00305 (0.00039)
		Poços	17	2	−1.069	3	0.324 (0.136)	0.00038 (0.00017)
		Quadrilátero	34	8	0.833	7	0.831 (0.036)	0.00313 (0.00019)
RPL3	K80 + I	Total	176	47		24	0.831 (0.016)	0.02907 (0.00049)
		Canastra	70	29	−0.802	14	0.776 (0.035)	0.00120 (0.00193)
		Poços	36	24	0.110	6	0.432 (0.099)	0.01176 (0.00323)
		Quadrilátero	70	29	0.032	8	0.376 (0.072)	0.01199 (0.00282)
POMC	HKY + I	Total	176	9		12	0.728 (0.001)	0.00382 (0.00023)
		Canastra	70	9	1.070	10	0.793 (0.031)	0.00437 (0.00028)
		Poços	36	7	1.224	8	0.816 (0.038)	0.00402 (0.00035)
		Quadrilátero	70	7	0.200	6	0.513 (0.005)	0.00261 (0.00045)

### 2.6 Ecological Niche Modeling (ENMs)

Using the bioclimatic data at a 2.5‐min resolution of latitude and longitude, we modeled species niche and projected distribution of *P. ayeaye* across the mountaintops of a selected region in southeastern Brazil. Because we had many more collection records than sample points (Table S1), we used the putative population limits estimated by geneland to assign known records to our delimited ESUs (Fig. S2). We obtained the climate layers from Hijmans, Cameron, Parra, Jones, and Jarvis (2005) to characterize the environmental space for ENMs using both present (1960–1990) and future climate conditions (average predicted for 2061–2080). The future climate layers are derived from three coupled Atmosphere‐Ocean General Circulation Models (AOGCMs): CCSM4, CNRM‐CM5, and MIROC5. We selected four variables (temperature annual range, mean temperature of warmest quarter, precipitation of wettest, and driest quarter) of 19 bioclimatic variables using a factorial analysis with a varimax rotation (implemented in psych package in r; available at https://CRAN.R-project.org/package=psych) (Table S2). This method is based on the correlation matrix among variables to minimize collinearity problems, consequently avoiding biased predictions of the ENMs.

A key assumption in our modeling is that ensemble forecasts based on multiple models generate more accurate or at least more conservative projections of species distribution than single models do (Araújo & New, 2007). Therefore, four modeling methods were used to build the ENMs, including Bioclim, Gower Distances, Maximum Entropy (MaxEnt), and Support Vector Machine (SVM), both presence‐only or presence background methods, and adequate to our limited data by its simplicity in the configurations (for a review, see Peterson et al., 2011). All ENMs were implemented in the dismo package in r (available at https://CRAN.R-project.org/package=dismo). In summary, models were first generated for present climate and then projected onto future conditions to predict the species geographic range for these two distinct time periods. We assessed model performance for each decision threshold using the “leave‐one‐out test” because of the small number of occurrence records for *P. ayeaye* and ESUs (Table S1). Hence, multiple predictions were made for *P. ayeaye* and ESUs, selecting one occurrence record for removal in each case. This approach is described as a variation to the k‐fold partitioning method on which a Jackknife sampling is imposed (Bean, Stafford, & Brashares, 2012; Pearson, Raxworthy, Nakamura, & Peterson, 2007; Shcheglovitova & Anderson, 2013). For each prediction, we applied the lowest presence decision threshold (LPT) to test the ability to predict the deleted occurrences. If the ENM successfully predicts both a small area and the deleted occurrence record, it is better than a random model (*p* < .05). However, if the model predicts a large area and fails to predict the deleted occurrence record, it is not considered a good model (*p* > .05). Therefore, the *p*‐value is calculated from the success and failure ratio of the prediction (Pearson et al., 2007).

Our modeling procedure resulted in combined suitability maps of the best models for *P. ayeaye* and ESUs, and for each climate condition (4 ENMs* occurrence records, for present climate and 4 ENMs * 3 AOGCMs for future climate condition * occurrence records, for future conditions). Finally, we obtained a consensus map for each combination of ENMs and AOGCM. To assess individual model variability sources, we separated and mapped uncertainties in forecast ensembles (Diniz‐Filho et al., 2009). For this purpose, we performed a two‐way ANOVA for each grid cell using suitability as response variable and the methodological components (AOGCMs and ENMs) as explanatory variables.

### 2.7 Effectiveness of PAs to ESUs protection

We were interested in verifying the PAs effectiveness to protect the entire *P. ayeaye*'s suitable area and each ESU separately. We overlapped the PAs with suitable areas in current time, and we also built future projections for the entire species and each of the ESUs. The suitable areas in the present and future were overlapped with layers of protected areas of integral protection (IUCN Categories I to IV) already established in the region. The rate of protected areas was calculated in relation to the entirety of the suitable areas. The layers of protected areas were obtained from The World Database on Protected Areas (available at https://www.unep-wcmc.org/resources-and-data/wdpa). To obtain insights on management alternatives (like corridors and translocation routes planning), we used the estimated models to test the niche overlap hypothesis between ESUs, assuming that ESUs with more similar niches should be candidates to improve connectivity. The applied tests were I statistic (*I*; Warren et al., 2008) and the relative rank (*RR*; Warren & Seifert, 2011), all made in enmtools v1.4.4 (Warren, Glor, & Turelli, 2010).

## 3 RESULTS

### 3.1 ESUs characterization and relationships

**Figure 3:**
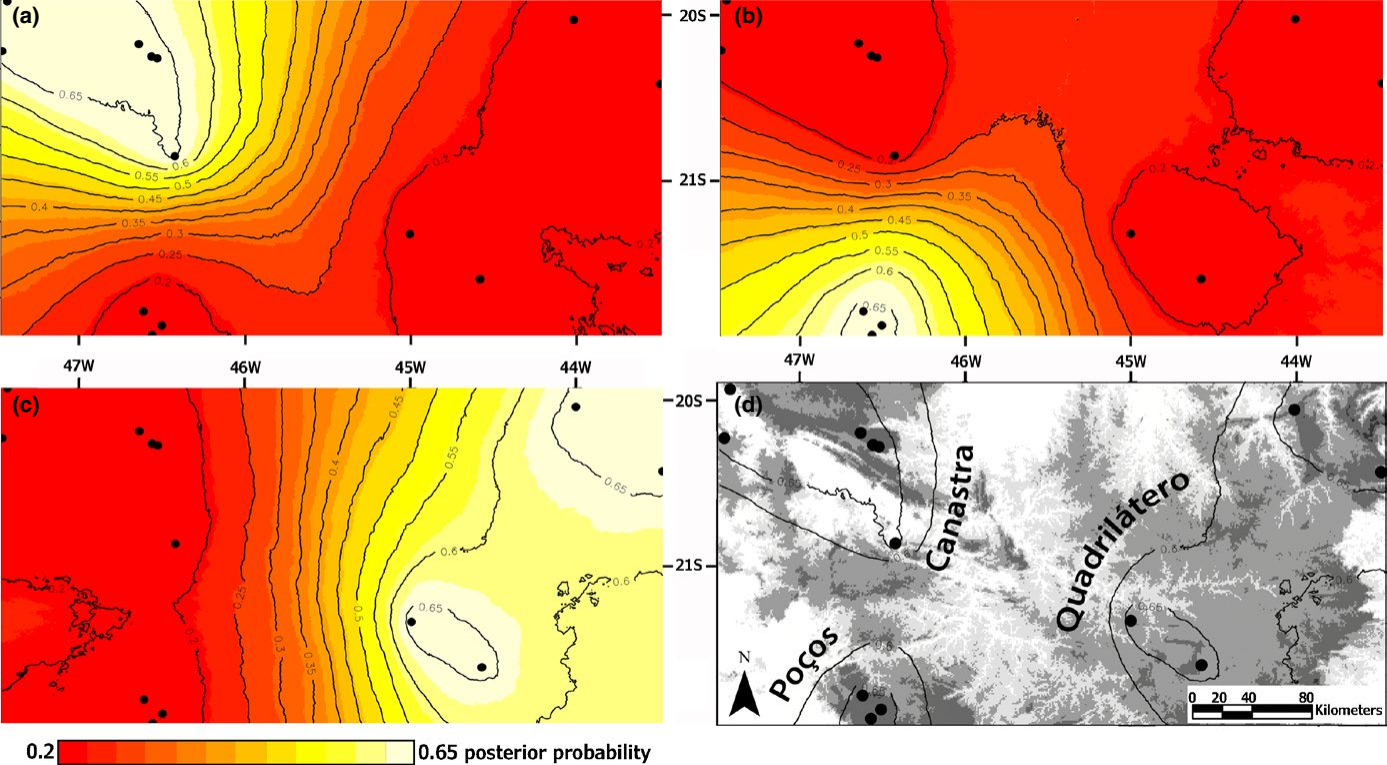
Maps of (a) Canastra, (b) Poços, and (c) Quadrilátero biogeographical units (BUs) membership posterior probabilities (PP). The geographic distribution of each BU is shown in (d), where the internal and external curves indicate geographic limits with PP = .65 and .6, respectively. The color scale of probabilities refers to a, b, and c. Gray scale in d refers to altitude, with darker colors indicating higher altitudes

**Figure 4:**
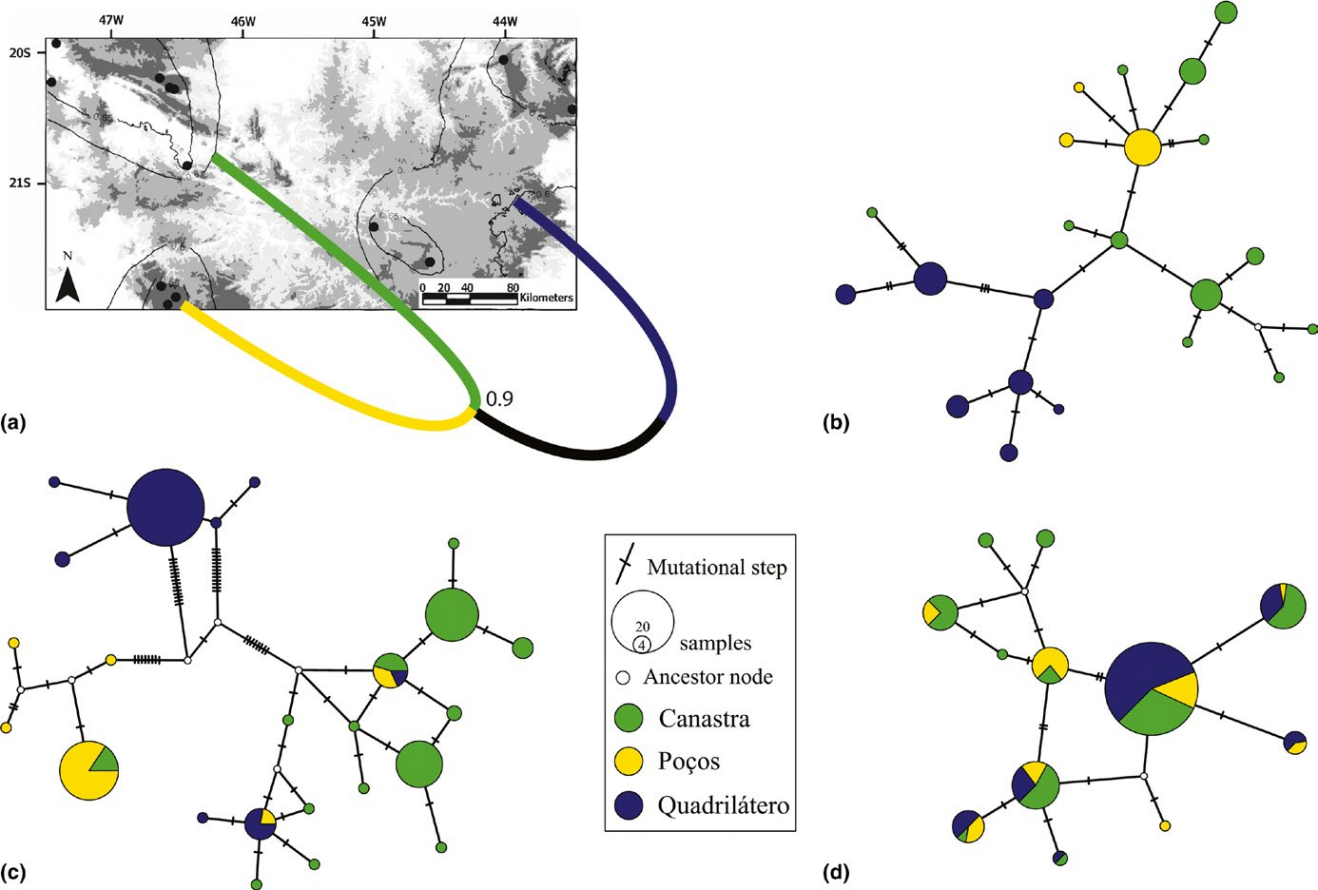
(a) Topology of the maximum credibility lineage tree showing genealogical relationship between biogeographic units. Number in node is Bayesian posterior probability. Statistical parsimony haplotype networks from (b) Cytochrome b, (c) intron 5 of Ribosomal protein L3, and (d) Proopiomelanocortin

We found correlation between genetic structure in *P. ayeaye* and mountain ranges where it occurs. Geneland runs showed convergence in posterior probabilities (PP) of models, after a burn‐in of 1 × 10^3^ replicates. The analyses returned three BUs with PP = .47 (Fig. S3). The geographic distribution of these lineages corresponded to the following mountain ranges: (1) Canastra Plateau and the surrounding mountains; (2) Poços de Caldas Plateau, southwest of the Mantiqueira Range; and (3) Quadrilátero Ferrífero plus the Southern Minas Gerais Mountains, named hereafter Canastra, Poços and Quadrilátero, respectively (Figure 3). The relationship between geographic and genetic distances can be explained by chance (*r* ^2^ = .243, *p* = .999), and thus, the lack of significant IBD reinforces the hypothesis that the genetic structure of *P. ayeaye* is related to isolation in sky islands. The lineage tree showed a closer relationship between Canastra and Poços as sister BUs (Figure 4a). The estimated mean substitution rates for POMC and RPL3 were 0.0043 (*SD* = 0.0021) and 0.0087 (*SD* = 0.0029) per lineage per million years, respectively.

The demographic model selection confirmed each BU as a distinct ESU, but with gene flow among them. The best island model was the one where Canastra and Quadrilátero are isolated from each other, with PP = .66 (Table 2; Fig. S1, model 3). The estimated values from *ϴ* and *M* from the best model are shown in Table 3. The model where Poços and Quadrilátero are isolated one from another (Fig. S1, model 2) had PP = .33 (Table 2). This non‐negligible probability for an alternative model may be due to incongruence among loci. While Cyt‐*b* and RPL3 show 76 and 96% of probability associated with model 3, respectively (Fig. S1), the best fit model for POMC was number 2, with 43% of probability (Fig. S1). In any case, our results suggest higher gene flow between the sisters ESUs of Canastra and Poços, but limited gene flow between Quadrilátero and at least one of the other ESUs. Furthermore, models of island isolation and panmixia had no support in either single‐ or multilocus analyses (Table 2). From the genetic point of view, the three BUs are ESUs from a historical metapopulation with a stepping‐stone island pattern among them.

**Table 2:** Model selection with migrate‐N. Parameter shown are model numbers, sorted by probability, equivalent to Fig. S1

Model	Description	Bezier's approximation score	PP	Weight
3	Canastra and Quadrilátero are isolated each other	−4266.55	.661	1
2	Poços and Quadrilátero are isolated each other	−4267.23	.335	0.507
1	All populations are interconnected	−4271.73	.004	0.006
6	Poços and Canastra form a panmictic population connected with Quadrilátero	−4333.22	<.001	<0.001
4	Poços and Canastra interconnected but isolated from Quadrilátero	−4495.11	<.001	<0.001
8	Panmixia	−4498.78	<.001	<0.001
5	All populations isolated each other	−4557.04	<.001	<0.001
7	Poços and Canastra form a panmictic population isolated from Quadrilátero	−4613.54	<.001	<0.001

PP is posterior probability.

**Table 3:** Comparison between estimated parameters using full Bayesian (starbeast2 and migrate‐N) and ABC approaches. Parameters shown are divergence times in million years (τ, assuming one generation per year), population sizes (ϴ), and effective number of migrants (M) of ESUs Canastra (C), Poços (P), and Quadrilátero (Q). τ in full Bayesian was estimated in starbeast2, while the remaining parameters were estimated in migrate‐N

Parameter	Full Bayesian		ABC	
	Mean	95% HPD	Mean	95% HPD
τ_1_	0.06845	0.01842–0.19665	0.07494	0.06894–0.08104
τ_2_	0.1089	0.02299–0.32562	0.3899	0.37569–0.40567
ϴ_C_	0.00376	0.00072–0.00391	0.00889	0.00884–0.00894
ϴ_P_	0.00129	0.00001–0.00182	0.0011	0.00100–0.00120
ϴ_Q_	0.00176	0.00066–0.00290	0.00138	0.00106–0.00167
M_C > P_	49.96	0.00–50.93	88.85	86.42–91.65
M_P > C_	13.04	0.00–44.27	40.52	37.46–44.34
M_P > Q_	9.48	0.00–37.61	83.41	79.86–87.19
M_Q > P_	15.14	0.00–42.94	72.66	70.99–74.68

Regarding ABC estimates, the evaluation of model fit done with a PCA analysis of summary statistics suggested that the simulated demographic model fits well the empirical data because the cloud of posterior data points matches the observed data point (Fig. S4). As expected, the divergence times estimated with the ABC approach (which takes gene flow among ESUs into account) were older than the starbeast2's estimates, especially the older splitting event, which was about 3.5 times larger on average (Table 3). Furthermore, *ϴ* estimates had values within the 95% highest posterior density (HPD) confidence intervals of migrate‐n parameters, except for Canastra's *ϴ* (Table 3). On the other hand, all migration rates were higher than those estimated in the full Bayesian approach (Table 3). All Cyt‐*b* haplotypes were reciprocally exclusive in each ESU, although they did not represent distinct haplogroups in the network, which is probably due to ILS or historical gene flow (Figure 4b). Despite the lack of a clear differentiation among ESUs in the POMC network (Figure 4d), the RPL3 network did exhibit divergence among them (Figure 4c), further supporting each BU as an ESU.

The Canastra ESU exhibited much more genetic diversity than the others (Tables 1 and 3). This does not seem to be a sampling artifact because Quadrilátero ESU has a similar sampling in terms of field work effort and geographic coverage (Table S1, Figure 3). The RPL3 marker presented excess of haplotype pairwise differences due to divergence between geographically divided ESUs (Figure 4c), further strengthening our BUs as ESUs (Tables 1 and 3).

### 3.2 ENMs and effectiveness of PAs to ESUs protection

**Figure 5:**
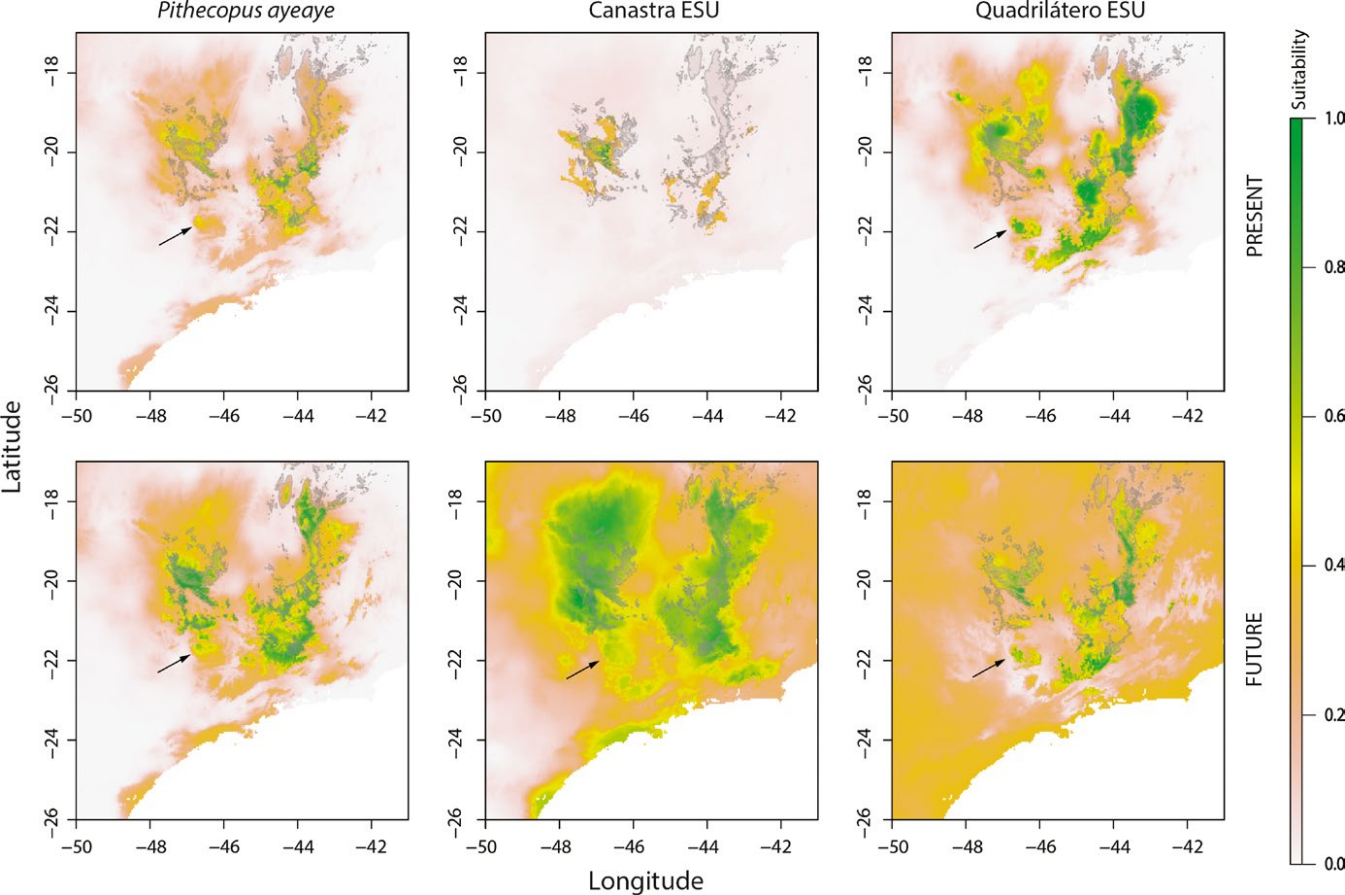
Potential geographic distribution for *Pithecopus ayeaye* B. Lutz, 1966, Canastra and Quadrilátero ESUs for both present and future climate conditions. Arrows indicate the Poços de Caldas Plateau region. Gray borders indicate the *campos rupestres* limits (modified from Fernandes et al., 2014)

**Figure 6:**
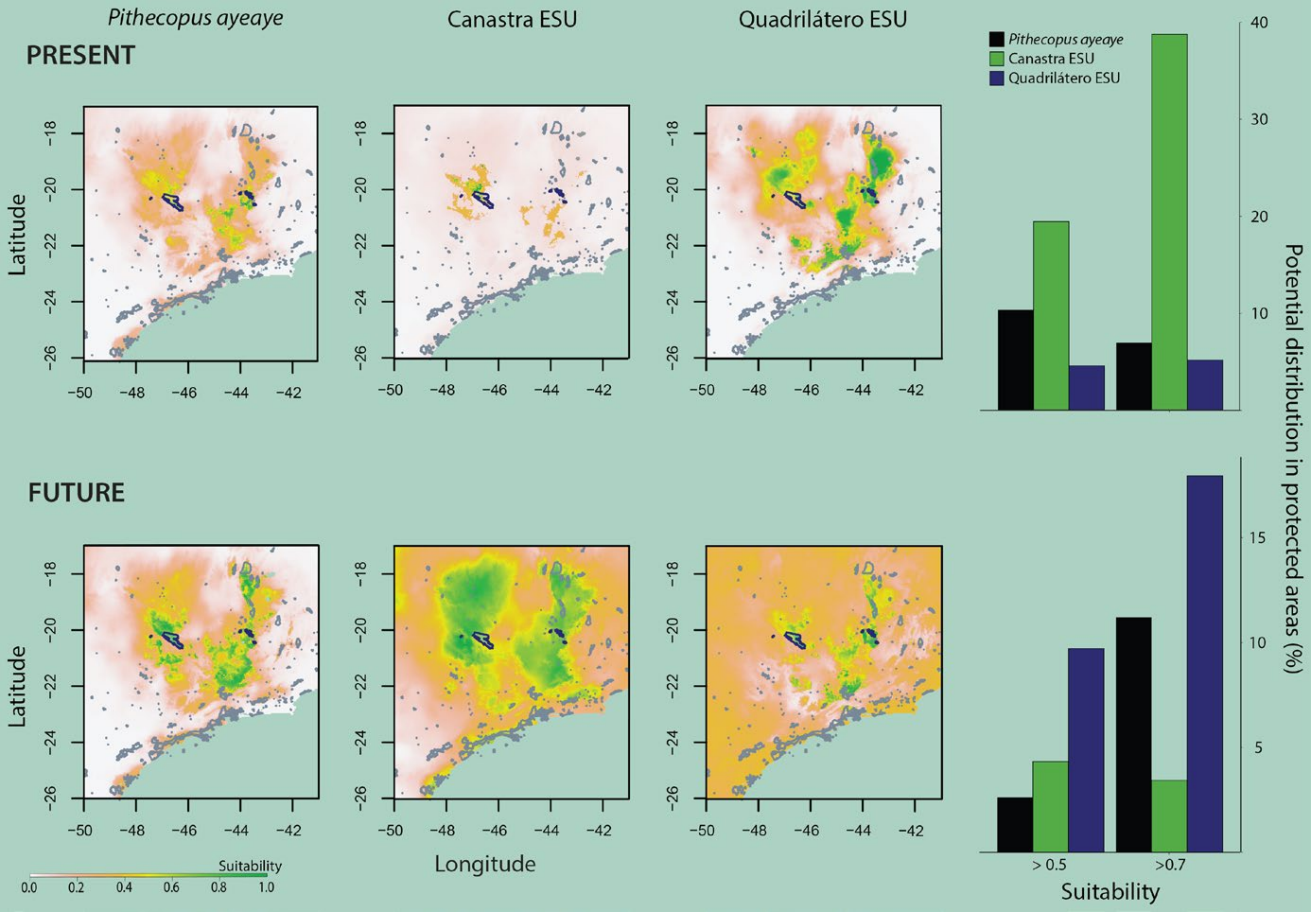
Left, protected areas (PAs) network overlapping the potential geographic distribution of *Pithecopus ayeaye* B. Lutz, 1966 and its ESUs. PAs where the species is known are highlighted in blue. Right, effectiveness of PAs network in protecting the species and its ESUs

We used the four ENM methods to build a consensus map of the projected present and future potential distribution for entire *P. ayeaye*, Canastra and Quadrilátero ESUs (Figure 5). Poços ESU was not included due to the scarcity of occurrence data to construct reliable models (Table S1). The projections presented for *P. ayeaye* and BUs were trained using between seven to 26 localities and show high success rates in Jackknife tests (Table 4). Under present climate conditions, the potential distribution of *P. ayeaye* occurred throughout mountaintops with a relatively high suitability (>0.5), mainly in the State of Minas Gerais, but with a predicted expanded distribution in future climate conditions. The potential distributions of Canastra and Quadrilátero ESUs are both locally suitable in the current climate conditions (Figure 6). However, there are clear changes in the patterns of suitability areas in future climate conditions, characterized by expansions to the north and south in relation to the present suitable distribution.

**Table 4:** Jaccknife test of distribution models for *Pithecopus ayeaye* B. Lutz, 1966 and ESUs for each ENMs applied

AOGCM^a^	ENM	*Pithecopus ayeaye*			Canastra ESU			Quadrilátero ESU		
		Sample Size	Success	*p*‐Value	Sample Size	Success	*p*‐Value	Sample Size	Success	*p*‐Value
Current	Bioclim	26	19	4.75E‐14	7	2	1.06E‐2	16	11	8.77E‐18
	Gower Distance	26	22	5.94E‐15	7	4	3.51E‐05	16	14	5.05E‐21
	Maxent	26	25	2.24E‐14	7	6	6.40E‐4	16	15	9.35E‐15
	SVM	26	25	3.16E‐17	7	6	4.75E‐05	16	15	2.33E‐24
CCSM4	Bioclim	26	9	1	7	0	1	16	0	1
	Gower Distance	26	25	1.04E‐18	7	4	6.10E‐4	16	13	8.07E‐19
	Maxent	26	25	1.47E‐15	7	6	4.80E‐4	16	15	1.24E‐19
	SVM	26	25	3.12E‐08	7	6	1.42E‐3	16	16	7.82E‐15
CNRM‐CM5	Bioclim	26	0	1	7	0	1	16	0	1
	Gower Distance	26	16	1.43E‐08	7	0	1	16	0	1
	Maxent	26	25	2.59E‐17	7	6	1.31E‐3	16	15	8.46E‐13
	SVM	26	25	1.23E‐08	7	6	6.98E‐3	16	14	3.81E‐19
MIROC5	Bioclim	26	1	1	7	0	1	16	0	1
	Gower Distance	26	22	6.71E‐15	7	2	4.75E‐3	16	3	5.58E‐3
	Maxent	26	25	1.05E‐16	7	6	3.67E‐04	16	15	7.62E‐12
	SVM	26	25	2.50E‐11	7	6	3.06E‐04	16	15	2.56E‐2

a Current refers to average of the climatic conditions between 1969 and 2000. The other AOGCM are future models.

The two‐way ANOVA applied shows that the median of the variation projected in future climate conditions for *P. ayeaye* and Quadrilatero ESU are due to differences in ENMs (up to 85.6%; Table 5), with the lowest differences in the southern limit of Minas Gerais State (Fig. S4). Furthermore, the maps showing this sum of squares (Fig. S5) indicate the largest differences among methods for *P. ayeaye*, Canastra and Quadrilatero ESUs. When considering the ENMs, both *P. ayeaye* (all populations) and individual ESUs can be potentially found in other PAs, in addition to those already known to protect the species. However, <15% of all suitability areas (even in more relaxed cutoff) are in protected areas for present projections of the species (Figure 6). For the Canastra ESU, more than 35% of highly suitable areas are within PAs, but this is drastically reduced in future conditions (~4%) (Figure 6). Even those models predict wide suitable climatic areas in PAs for the current climate, the known occurrence present only in rock outcrops should prevent its expansion in future climate conditions. From an ecological perspective, Canastra and Quadrilátero populations do not present identical niches, but show great overlap (*I *=* *0.84 and *RR *= 0.86). In relation to the global model (that includes all occurrence points of the species), the ESUs show almost complete overlap (Canastra: *I *=* *0.96 and *RR *= 0.92; Quadrilátero: *I *=* *0.9 and *RR* = 0.91). For these reasons, and for conservation management purposes, the climate conditions between the ESUs are indistinguishable. Although the Poços ESU was not included in niche overlap tests, it is important to note that the Quadrilátero niche model comprises the area of Poços de Caldas Plateau with a high probability.

**Table 5:** Median proportions of the total sum of squares from the two‐way anova performed for each grid cell covering the Neotropics, evaluating the relative contributions of method for niche models and Atmospheric‐Ocean Global Circulation Models (AOGCM) to the variability in forecasting *Pithecopus ayeaye* B. Lutz, 1966 and ESUs distribution. Minimum and maximum values in the maps are also given (see also Fig. S5)

Source	*Pithecopus ayeaye*		Canastra ESU		Quadrilátero ESU	
	SS (%) median	Min‐max	SS (%) median	Min‐max	SS (%) median	Min‐max
ENMs	0.898	0.001–0.999	0.897	0–1	0.856	0.003–0.999
AOGCM	0.033	0–0.914	0.033	0–0.914	0.045	0–0.722
ENMs x AOGCM	0.06	0–0.886	0.061	0–0.886	0.087	0–0.764

## 4 DISCUSSION

Our results show that the *P. ayeaye* displays a genetic structure associated with the spatial fragmentation of a sky island environment. Moreover, despite the reported wide range of *P. ayeaye* (Baêta et al., 2009), it is known to occur only in five PAs currently: both Furnas do Bom Jesus State Park and Serra da Canastra National Park protecting the Canastra ESU, as well as Itacolomi State Park, Serra do Rola Moça State Park and Gandarela National Park protecting the Quadrilátero ESU (Araujo et al., 2007; Baêta et al., 2009; FSFL, personal observation). Together, those five PAs encompass <4% of the entire, potential and suitable geographic distribution of *P. ayeaye*. In the future scenario, with the predicted expansion of the species, the coverage of protected areas for *P. ayeaye* tends to worsen or to improve unsatisfactorily, depending on suitability cutoff (Figure 6). To aggravate the situation, *P. ayeaye* exhibits a set of life history traits associated with vulnerability in amphibians. Its rarity, *K*‐reproductive strategy, and high habitat specialization are characteristics associated with low genetic diversity, increased population structure, and/or high threat levels (Cooper et al. 2008; Romiguier et al., 2014; Toledo, Becker, Haddad, & Zamudio, 2014; Rodríguez et al., 2015). Indeed, the smallest genetic diversity was observed in Poços and Quadrilátero ESUs, which may be associated with increased risks of local extinction. As *P. ayeaye* displays a naturally fragmented distribution of populations with evolutionary independence and is endemic to Brazil, we suggest that the conservation status of the species should be revised in the national list of endangered species because its long‐term survival depends on local policies. For accurate recategorization, more research on demographic size and population trends needs to be conducted.

Our protocol to identify ESUs allowed us to validate *P. ayeaye*'s ESUs even with the lack of reciprocal mtDNA monophyly (Moritz, 1994). Moreover, the ESUs exhibited exclusivity in mtDNA haplotypes, meeting the requirements proposed by the Fraser and Bernatchez's ESU definition. This idea may be applied with any coalescent sampler that implements model selection (see Carstens, Brennan et al., 2013), not being limited to the use of migrate‐N. Additionally, if there is no evidence of gene flow between ESUs, a coalescent method of species delimitation may be subsequently applied to distinguish between ESU and distinct species with any multilocus sequence dataset (Carstens, Pelletier, et al., 2013). Geographically isolated populations may experience cyclical events of gene flow, especially in those species associated with interglacial refugia (Bonatelli et al., 2014). Gene flow breaks differentiation between lineages and generates mtDNA para‐ or polyphyly, a condition that does not meet the requirements of Moritz's (1994) definition of ESU. The τ estimates indicate recent diversification events between Middle and Late Pleistocene, which is congruent with the lack of well‐defined haplogroups in the Cyt‐*b* network. A varying level of haplotype or haplogroup sharing, as we observed between the ESUs, is expected as a result of historical gene flow before the more recent divergence event (Pinho & Hey, 2010). Therefore, the distribution pattern of *P. ayeaye* could be associated with interglacial refugia, a pattern that may be common in endemic species of *campos rupestres* (Bonatelli et al., 2014). This raises the hypothesis of potential gene exchange during historical phases of expansion and recontact between ESUs. Nevertheless, more detailed phylogeographical studies should be carried out to test this hypothesis, because knowledge about past distribution dynamics can provide insights for improved conservation decisions (Forester et al., 2013). Our results also suggest that the ESUs’ delimitation method suggested by Moritz (1994) may be too restrictive, and more relaxed criteria (but not less methodologically rigorous) could be applied to phylogeographical data in order to accommodate cases of recent diversification and/or repeated recontact among formerly isolated lineages.

Despite this, the *campos rupestres* is expected to contract in warmer climatic conditions (Bonatelli et al., 2014; Fernandes et al., 2014), and our results show a surprising potential future expansion for the species’ distribution. Thus, we can conclude that the major threats to the species are more related to habitat loss due to environmental degradation than to climate changes. The streams where the species breeds are extremely susceptible to erosion due to steep slopes and superficial soil. Currently, cattle grazing and degradation of marginal vegetation are the main threats of these fragile environments (e.g., Brandão & Álvares, 2009). In a more distal analysis, if climate changes lead to landscape structure alterations with negative impacts on stream rivulets, *P. ayeaye* could even be more threatened, even though our ENMs show an optimistic scenario for the species.

The niche overlap analysis indicates no climate selection between ESUs, which allows us to suggest conservation strategies that may be implemented in the present with potential positive impacts in the future. In situ conservation is the most feasible alternative for amphibian protection in Brazil (Haddad, 2008). Therefore, the expansion of the Brazilian PAs network in mountaintops where *P. ayeaye* occurs, taking into account its intraspecific diversity, can mitigate the impacts suffered by the species, and consequently, other endemic, co‐occurring organisms. Another strategy to avoid genetic diversity loss and inbreeding could be the translocation of individuals between ESUs (Weeks et al., 2016). Our results provide valuable information to plan this strategy, suggesting that individuals from the Canastra lineage could eventually be translocated to the Poços de Caldas Plateau, to increase the genetic diversity in the Poços ESU. However, this strategy should be done carefully to avoid possible negative consequences, such as outbreeding depression (see Frankham et al., 2011). The lack of knowledge regarding the species’ dispersal capacity between the sky islands at present has led us to conclude that there is an unviability, or an uncertainty, as to whether the implementation of corridors between the mountaintops would function. Even though the maintenance of evolutionary potential is important for allowing amphibians to cope with environmental changes (Allentoft & O'Brien, 2010), few countries take intraspecific diversity into account for their conservation policies, and Brazil is unfortunately not among them. For example, the detection of three remarkably divergent ESUs in the Atlantic Forest sloth, *Bradypus torquatus* (Lara‐Ruiz, Chiarello, & Santos, 2008), was not enough to avoid downlisting the species from Endangered to Vulnerable (Brazilian List of Endangered Species and IUCN). Most *P. ayeaye* ESUs lack effective protection because areas with integral protection only cover a small portion of their present distribution. Although the implementation of protected areas of sustainable use for *P. ayeaye* protection may seem a viable alternative, these categories are inefficient to avoid habitat loss in the Brazilian Cerrado (Françoso et al., 2015), which would not guarantee the species protection in the future, especially because some of them allow cattle raising. Additionally, many areas lack any kind of protection, as the species type locality in Morro do Ferro in Poços de Caldas, which is the ESU with one of the smallest genetic diversities in our results. The creation of an integral protection PA in this area must be a priority, as another endangered species, such as the *Bokermannohyla vulcaniae* and *Proceratophrys palustris*, are only known to occur in this region (ICMBio, 2014).

Notwithstanding, our study has its own caveats. The locality points were mainly defined by opportunistic sampling of literature and voucher specimens from museums and collections. Thus, our ENM analyses based on low sample sizes with a likely sampling bias (*i.e.,* collected in shrubs near pools or rivulets) can underestimate the species occurrence with consequences to model accuracy and interpretability (see Peterson et al., 2011). Many studies have shown that model accuracy using about 30 occurrences is often low, and is quite heterogeneous across species (i.e., Hernandez, Graham, Master, & Albert, 2006; Wisz et al., 2008), even though other studies have adjusted the ENMs for fewer occurrences (i.e., Pearson et al., 2007; Shcheglovitova & Anderson, 2013). Furthermore, we assumed the distribution of the species and its ESUs are delimited by climate. However, *P. ayeaye* is clearly a *campos rupestres* endemic, and our models also predicted the species occurrence in the Atlantic Forest and areas of the Cerrado savannah. Therefore, the occurrence areas of the species should be significantly more restricted. Moreover, the ENMs predicted the occurrence of *P. ayeaye* in the southern region of the Espinhaço Meridional and Serra do Cabral, areas where only *P. megacephalus*, another *campos rupestres* endemic, was registered. Thus, some PAs like Serra do Cipó National Park, Serra do Cabral State Park, and Sempre Vivas National Park were included in our protected areas account. Whether by competition or by historical contingency, there is no sympatry record between the two species. With the removal of these PAs, the rate of distribution of *P. ayeaye* covered by the PA network becomes even more alarming. In relation to the geneland analysis, some localities had few samples. It is known that small sample sizes can reduce the accuracy of Bayesian clustering methods (Corander, Waldmann, & Sillampaa, 2003). For example, concerning the Pedregulho and Alpinópolis sample points from the southern river‐side of Rio Grande valley, there are only three samples and this large river may be a possible barrier to gene flow. Thus, we cannot rule out that increased sampling at this region might reveal another ESU. Even with these limitations, this study is important for providing valuable data about a species whose conservation status shows inconsistencies between global and national red lists. Finally, we used three independent sequence markers to make inferences about intraspecific diversification and relationships between *P. ayeaye* lineages*. *However, the use of a larger number of markers, like microsatellites and single nucleotide polymorphisms, should increase statistical power for clustering analysis and gene flow estimates (Allendorf, Honehlohe, & Luikart, 2010).

Our study sheds new light on conservation practices for amphibians in Brazil, as we found significant divergence among *P. ayeaye* populations that define three ESUs associated with distinct mountain regions, including one ESU found exclusively in an area without any kind of protection. This population structure reflects also the patterns found in many other taxa of the *campos rupestres* such as endangered lineages of the cactus *Pilosocereus aurisetus* (Bonatelli et al., 2014), the near threatened shrub *Lychnophora ericoides* (Collevatti et al., 2009), and the anurans *Pithecopus megacephalus* and *Bokermannohyla saxicola* (RFM, FRS and PCAG, unpublished data). Both have an associated structure with mountain ranges or geological subdivisions within them, as in *P. ayeaye*. Therefore, common preservation strategies can be applied for many Brazilian mountaintop endemic species to ensure their future viability. To accomplish this goal, intraspecific studies of the Brazilian sky islands’ biota are greatly needed in order to guide decision‐makers in generating policies that consider evolutionary and ecological processes and specificities of this ecosystem.

## CONFLICT OF INTEREST

None declared.

## AUTHOR CONTRIBUTIONS

RFM, AC, PL and FRS designed the study; RFM, HT, RAB and FSFL collected samples; RFM, HT and FRS produced the molecular data; RFM, AC, PL and UO analyzed the data; RFM led and all authors contributed to the writing of the manuscript.

## DATA ACCESSIBILITY

DNA data are archived in GenBank (accession numbers MF158349–158608). Information on the samples and occurrence points used in this study is available in Supporting Information.
