# Supplementary material for: Evolutionarily significant units of the critically endangered leaf frog Pithecopus ayeaye (Anura, Phyllomedusidae) are not effectively preserved by the Brazilian protected areas network
Source: Ecol Evol. 2017 Sep 20;7(21):8812–28. doi: 10.1002/ece3.3261 (PMC5689491; doi:10.1002/ece3.3261)
Supplement: Supplementary file 1 [file ECE3-7-8812-s001.pdf]

Appendix S1. Voucher information on the individuals used in the study.

The voucher numbers were ***ESU Canastra:*** Alpinópolis – MG: AAG 510; Pedregulho – SP: CFBH-T 4388-89; Sacramento – MG: CFBH-T 1890-91, 11889-92, 12548-52, 19783-85; São Roque de Minas – MG: AAG 502, 588, 1265; CFBH-T 89, 153; CHUNB 51420-21, 56876; UFMG-A 16828-32, 16837, 16840; UFMG-G 1608 (two individuals), 1610, 1613 (two individuals). ***ESU Poços***: Poços de Caldas – MG: AAG 486; Délio Baêta field series 557 (to be deposited in CFBH-T); CHUNB 51410, 51412-18; UFMG-A 17115-17; UFMG-G 1837-40, 1844. ***ESU Quadrilátero:*** Lavras – MG: CHARW-T 1463; Minduri – MG: UFMG-A 16414-28, 16436-38; Nova Lima – MG: UFMG-A 14920, 18736; Ouro Preto – MG: UFMG-A 14925, 14978-80, 15420-24, 15426-29.

Table S1. Occurrence points of *Pithecopus ayeaye* . N = number of individuals, ESU = evolutionarily significant units.

Locality	N	ESU	Latitude	Longitude	Source
Sampled locations of vouchered individuals of *Phyllomedusa ayeaye* (B. Lutz, 1966) used in this study.					
Alpinópolis - MG	1	Canastra	-20.8597	-46.4203	AAG
Lavras - MG	1	Quadrilátero	-21.2013	-44.9413	CHRWT
Minduri - MG	18	Quadrilátero	-21.5934	-44.5719	UFMG
Nova Lima - MG	2	Quadrilátero	-20.0552	-44.0135	UFMG
Ouro Preto - MG	14	Quadrilátero	-20.4309	-43.4904	UFMG
Pedregulho - MG	2	Canastra	-20.2256	-47.4539	CFBH
Poços de Caldas - MG	18	Poços	-21.9172	-46.5686	UFMG
			-21.8612	-46.5086	UFMG
			-21.7772	-46.6192	UFMG
Sacramento - MG	12	Canastra	-19.9316	-47.4183	CFBH
São Roque de Minas - MG	20	Canastra	-20.2669	-46.5553	UFMG
			-20.1939	-46.6306	UFMG
			-20.2792	-46.5208	UFMG
Additional occurrence points of *Phyllomedusa ayeaye* (B. Lutz, 1966) used in ecological niche modelling.					
Arantina - MG	-	Quadrilátero	-21.8609	-44.2165	R.A.B.
Brumadinho - MG	-	Quadrilátero	-20.1014	-43.9888	UFMG
	-	Quadrilátero	-20.094	-44.021	UFMG
	-	Quadrilátero	-20.0837	-43.9951	UFMG
	-	Quadrilátero	-20.1016	-43.9884	R.A.B.
Carrancas - MG	-	Quadrilátero	-21.5939	-44.6272	R.A.B.
Congonhas do Campo - MG	-	Quadrilátero	-20.4536	-43.8742	UFMG
	-	Quadrilátero	-20.1278	-43.9835	UFMG
	-	Quadrilátero	-20.4381	-43.9398	UFMG
	-	Quadrilátero	-20.4867	-43.9431	UFMG
	-	Quadrilátero	-20.4334	-43.874	R.A.B.
	-	Quadrilátero	-20.4827	-43.9368	R.A.B.
Itabirito - MG	-	Quadrilátero	-20.2309	-43.8524	UFMG
	-	Quadrilátero	-20.2934	-43.9306	R.A.B.
Lavras - MG	-	Quadrilátero	-21.3277	-44.9797	UFMG
Luminárias - MG	-	Quadrilátero	-21.5535	-44.8187	R.A.B.
Minduri - MG	-	Quadrilátero	-21.5941	-44.5739	UFMG
Nova Lima - MG	-	Quadrilátero	-20.1015	-43.9886	UFMG
	-	Quadrilátero	-20.0051	-43.9281	UFMG
	-	Quadrilátero	-20.0083	-43.9309	R.A.B.
Ouro Branco - MG	-	Quadrilátero	-20.5143	-43.6197	UFMG
	-	Quadrilátero	-20.5089	-43.6155	UFMG
	-	Quadrilátero	-20.4797	-43.5931	MZUFV
	-	Quadrilátero	-20.5086	-43.6131	MZUFV
	-	Quadrilátero	-20.5149	-43.6262	R.A.B.
Ouro Preto - MG	-	Quadrilátero	-20.4323	-43.4917	UFMG
	-	Quadrilátero	-20.4315	-43.487	UFMG
	-	Quadrilátero	-20.4321	-43.4877	UFMG
	-	Quadrilátero	-20.2777	-43.5257	MZUFV
	-	Quadrilátero	-20.3333	-43.4833	R.A.B.
	-	Quadrilátero	-20.4777	-43.6874	R.A.B.
Poços de Caldas - MG	-	Poços	-21.7766	-46.6178	CFBH
	-	Poços	-21.9088	-46.5467	R.A.B.
	-	Poços	-21.8977	-46.547	R.A.B.
Pedregulho - SP	-	Canastra	-20.2148	-47.4264	R.A.B.
Sacramento - MG	-	Canastra	-20.2197	-47.1062	CFBH
São Roque de Minas - MG	-	Canastra	-20.2682	-46.5549	UFMG
	-	Canastra	-20.2283	-46.4564	R.A.B.
	-	Canastra	-20.2778	-46.5221	R.A.B.
	-	Canastra	-20.2977	-46.5244	R.A.B.
	-	Canastra	-20.2682	-46.5549	R.A.B.

Table S2. DNA divergence within and between biogeographical units for all markers. C. Canastra, P. Poços and Q. Quadrilátero

Fragment	π_{CP}	π_{CQ}	π_{PQ}
cyt-b	2.231	4.874	4.328
POMC	2.542	2.212	1.983
RPL3	16.778	21.3	21.974

Table S3. Loadings of the bioclimatic variables in the first five axes of Varimax Rotated Factor Analysis, based on current climate. Numbers in bold highlight the highest loading, and based on this highest value one variable per factor was selected.

Bioclimatic variables	I	II	IV	III	V
1	0.945	0.282	-0.127		
2	0.597	0.751	0.25		
3	0.186	0.823	0.171	0.462	
4	-0.351	-0.747	0.264	-0.468	
5	0.93	0.19	-0.226	0.179	
6	0.891	0.147	-0.144	-0.389	
**7**	-0.105	0.976	-0.103		
8	0.969	-0.171			
9	0.883	0.385	0.221		
**10**	0.973	-0.195			
11	0.9	0.38	-0.101	0.157	
12	-0.209	-0.416	0.858	-0.132	0.101
13	-0.138	0.381	0.879		
14	-0.192	-0.968	-0.112		
15	0.167	0.929	0.235	0.12	
**16**	-0.135	0.3	0.937		
**17**	-0.208	-0.972			
18	-0.32	-0.166	0.727		
19	-0.172	-0.958			
	I	II	IV	III	V
SS loadings	6.524	6.122	3.169	1.868	0.633
Proportion Var	0.343	0.322	0.167	0.098	0.033
Cumulative Var	0.343	0.666	0.832	0.931	0.964

1.Annual Mean Temperature; 2.Mean Diurnal Range (Mean of monthly (max temp - min temp)); 3.Isothermality; 4.Temperature Seasonality (standard deviation *100); 5.Max Temperature of Warmest Month; 6.Min Temperature of Coldest Month; 7.Temperature Annual Range; 8.Mean Temperature of Wettest Quarter; 9.Mean Temperature of Driest Quarter; 10.Mean Temperature of Warmest Quarter; 11.Mean Temperature of Coldest Quarter; 12.Annual Precipitation; 13.Precipitation of Wettest Month; 14.Precipitation of Driest Month; 15.Precipitation Seasonality (Coefficient of Variation); 16.Precipitation of Wettest Quarter; 17.Precipitation of Driest Quarter; 18.Precipitation of Warmest Quarter; 19.Precipitation of Coldest Quarter.

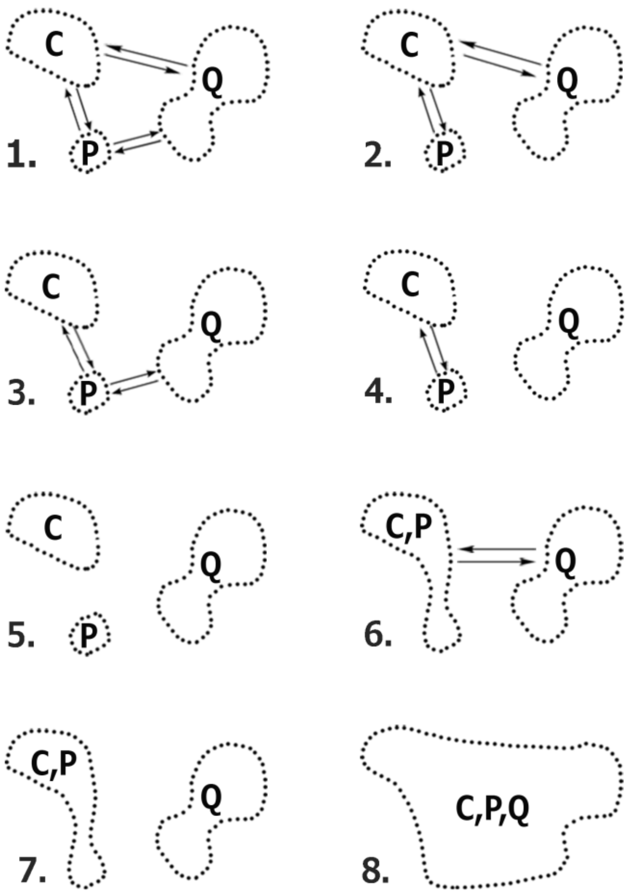

Fig. S1. Graphical representation of the eight-island hypothesis tested in MIGRATE-N model selection.

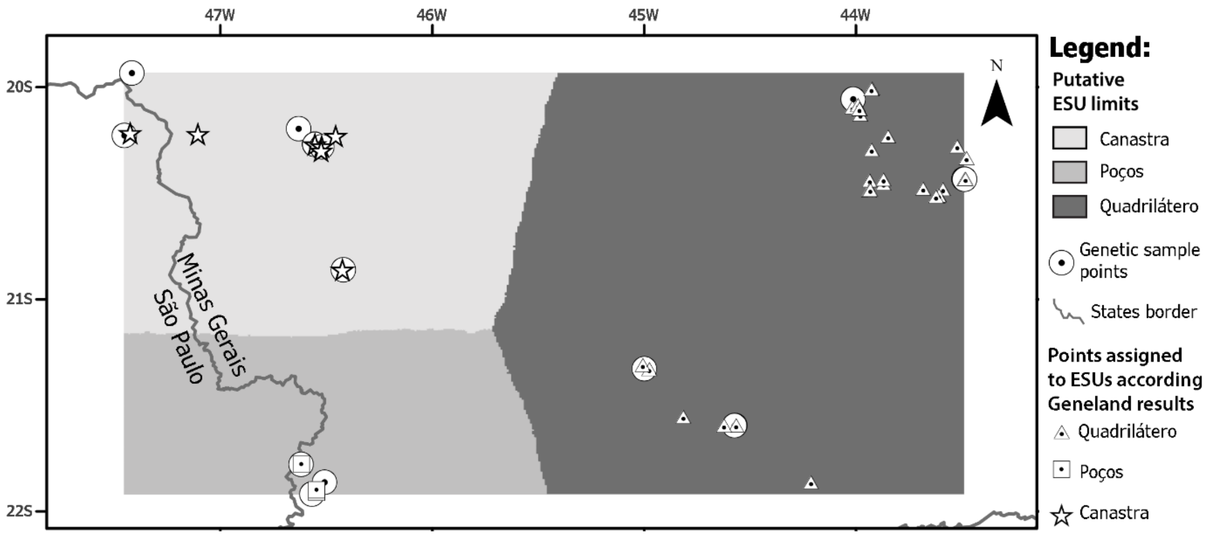

Fig. S2. Putative borders between biogeographical units (population limits) and sets of occurrence points used in ecological niche modelling.

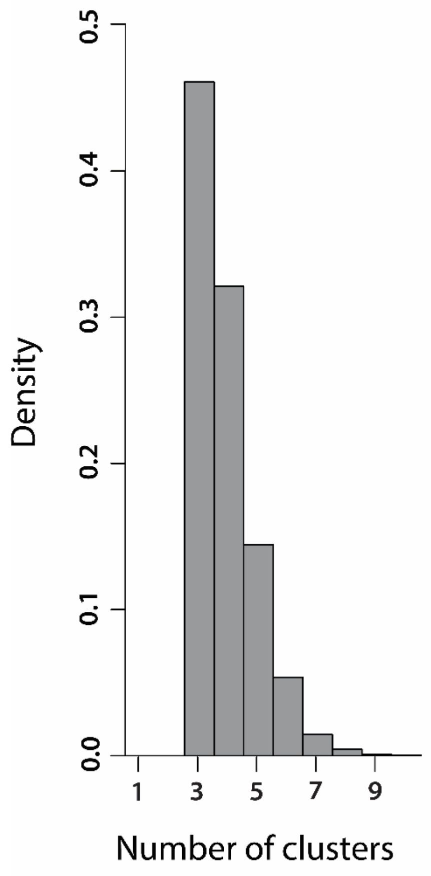

Fig. S3. Number of clusters along the chain after burn-in in GENELAND analysis.

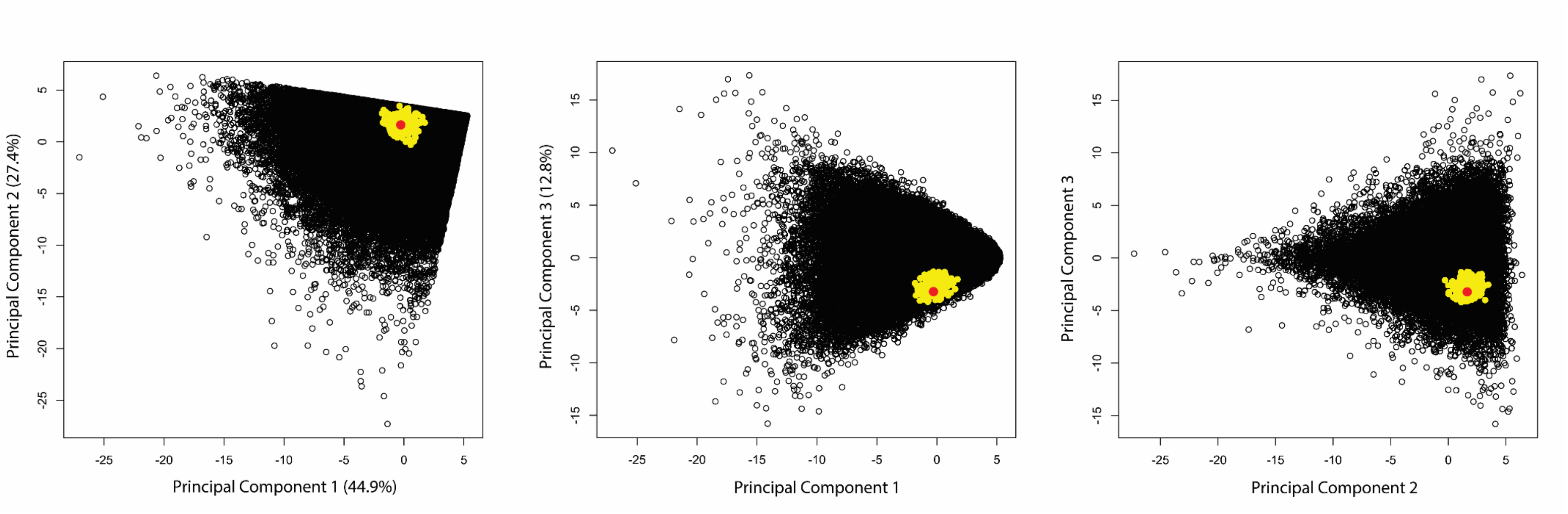

Fig. S4. Principal component analysis made to evaluate the performance of ABC results showing prior (black), posterior (yellow), and observed data (red).

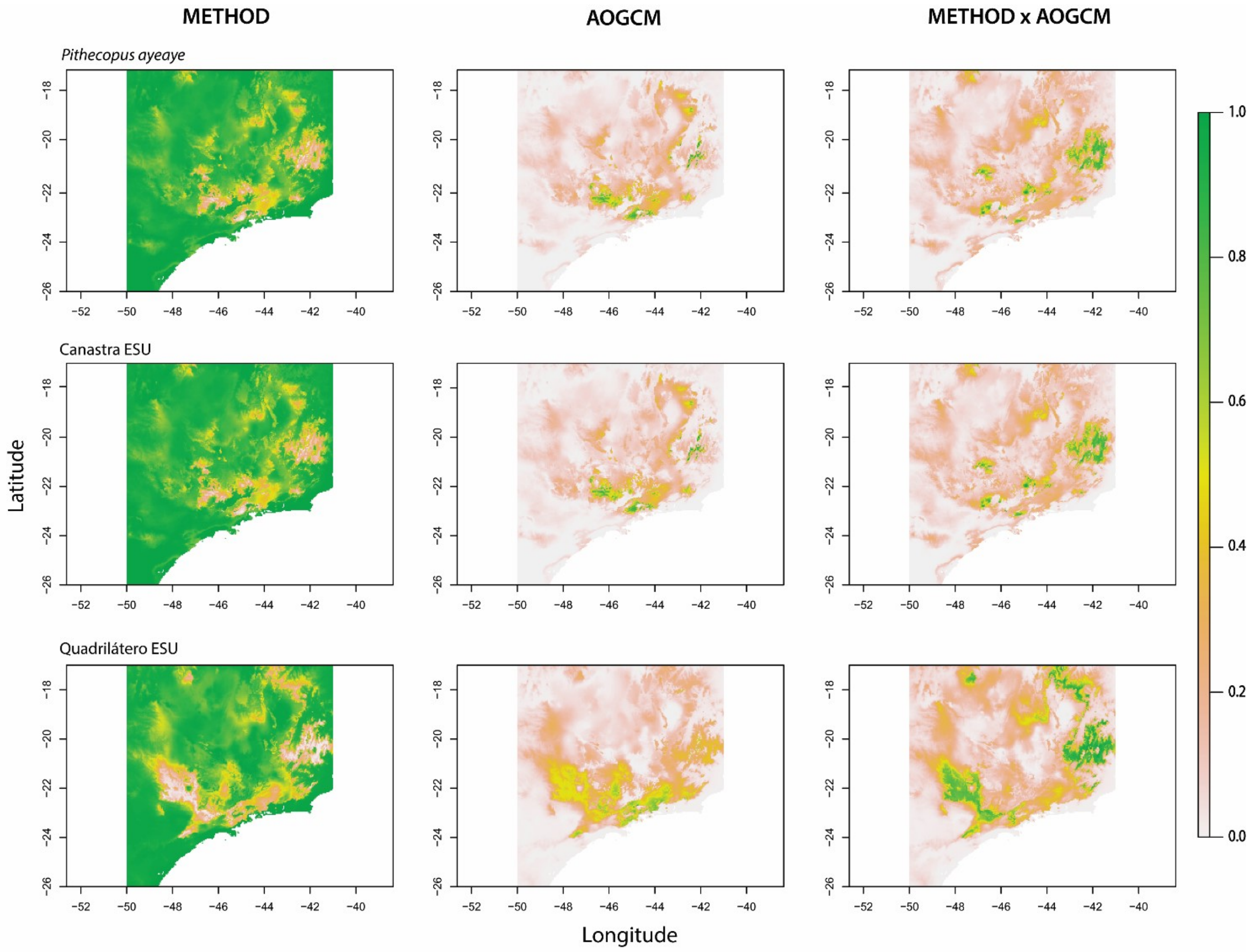

Fig. S5. Maps of variance (relative sum of squares) for the effect of ecological niche modelling (ENMs), Atmosphere-Ocean Global Circulation Models (AOGCMs), and the interaction between both sources of uncertainty for (A) *Pithecopus ayeaye*, and ESUs (B) Canastra and (C) Quadrilátero.
